# Sustainable Bio-Based Phenol-Formaldehyde Resoles Using Hydrolytically Depolymerized Kraft Lignin

**DOI:** 10.3390/molecules22111850

**Published:** 2017-10-28

**Authors:** Homaira Siddiqui, Nubla Mahmood, Zhongshun Yuan, Ferdinando Crapulli, Luana Dessbesell, Amin Rizkalla, Ajay Ray, Chunbao (Charles) Xu

**Affiliations:** 1 Department of Chemical and Biochemical Engineering, Faculty of Engineering, Western University, London, ON N6A 5B9, Canada; hsiddiq5@gmail.com (H.S.); nublamahmood@gmail.com (N.M.); zyuan25@uwo.ca (Z.Y.); fcrapulli@uwo.ca (F.C.); arizkalla@eng.uwo.ca (A.R.); 2 Faculty of Natural Resources Management, Lakehead University, Thunder Bay, ON P7B 5E1, Canada; ldessbes@lakeheadu.ca

**Keywords:** Kraft lignin, depolymerized Kraft lignin, phenol-formaldehyde resoles, curing temperature, optimization

## Abstract

In this study bio-based bio-phenol-formaldehyde (BPF) resoles were prepared using hydrolytically depolymerized Kraft lignin (DKL) as bio-phenol to partially substitute phenol. The effects of phenol substitution ratio, weight-average molecular weight (*M_w_*) of DKL and formaldehyde-to-phenol (F/P) ratio were also investigated to find the optimum curing temperature for BPF resoles. The results indicated that DKL with *M_w_* ~ 1200 g/mol provides a curing temperature of less than 180 °C for any substitution level, provided that F/P ratios are controlled. Incorporation of lignin reduced the curing temperature of the resin, however, higher *M_w_* DKL negatively affected the curing process. For any level of lignin *M_w_*, the curing temperature was found to increase with lower F/P ratios at lower phenol substitution levels. At 25% and 50% phenol substitution, increasing the F/P ratio allows for synthesis of resoles with lower curing temperatures. Increasing the phenol substitution from 50% to 75% allows for a broader range of lignin *M_w_* to attain low curing temperatures.

## 1. Introduction

Phenol formaldehyde (PF) resins are the second most common resins (following UF resins) in wood applications, and are in high demand in the manufacture of softwood plywood for exterior building and construction purposes [1,2]. The PF resin market is valued at $10 billion globally, and has an annual market value of $4.5 billion to $6 billion [3]. PF resins are known for their superior high bond strength and moisture resistance properties particularly under severe conditions of weather, temperature and humidity.

Phenolic resins are typically dark-reddish in color and can be synthesized at a variety of temperatures, ranging from room temperature to 135 °C. Curing temperatures range normally from 125 °C to 150 °C; however, hot-pressing temperatures for materials used in structural composites can reach up to 200 °C [1]. In the United States, PF resins are typically alkaline-catalyzed and are known as resoles. Application of resoles as wood adhesives became common around the 1930s, especially in the manufacture of particleboard and plywood [4]. Resoles are preferred for wood adhesives because of their ability to form three dimensional networks that have favorably high tensile strength, high modulus, dimensional stability, and resistance to moisture [4]. Resoles do not require the addition of a curing agent during the crosslinking stage and there is less concern for wood damage using base-catalyzed resins.

Typically, resoles are a mixture of oligomers of several units, residual free phenols and formaldehyde, as well as methylolated phenolic groups [4]. Production and application of PF resoles involve three reaction sequences, including formaldehyde addition to phenol, condensation reactions to form the resin, and finally curing of the polymeric resin upon application. The curing stage is characterized by more complex molecular rearrangements and reactions resulting from further condensation reactions at higher temperatures of above 125 °C. Resoles tend to cure without the addition of a curing agent, while novolacs require a curing agent for cross-linking of the network resin polymer. Beyond 170 °C, ether linkages may undergo further reactions to form newer arrangements such as methylene linkages that form with loss of formaldehyde. Above 200 °C, the resole undergoes thermal and oxidative decomposition of its structure as well as other complex reactions that may include further polymerization of the products.

Until now, around 95% of the phenol used in the production of PF resins is derived from petroleum products [5]. Due to depleting fossil fuel reserves as well as increasing environmental concerns as a result of accumulating greenhouse gases in the atmosphere, scientists and researchers have shown interest in replacing the phenol in PF resins with a renewable feedstock such as tannin, cashew nut shell, and lignin. Bio-based phenol formaldehyde (BPF) resins with lignin or lignin derivatives as substitute for phenol have been of particular interest given the wide availability of lignin as well as its low cost and low toxicity in comparison to petroleum derived phenol [6].

Among the renewable resources, lignin may be the best potential candidate for the production of PF resoles because of its aromatic structure. Lignin is composed of three different phenyl-propane monomer units including *p*-hydroxyphenylpropanol, guaiacylpropanol, and syringylpropanol predominantly bonded by aryl ether linkages [7]. Because of its complex structure; lignin has reduced reactive sites as compared to conventional phenols [6,8,9]. Owing to the presence of phenolic groups in the structure, lignin has the potential to replace phenol in the synthesis of PF resoles. The direct use of lignin in the production of PF resins requires longer press times as well as higher curing temperatures [10]. Recent studies have proposed the inclusion of pretreatment processes such as demethylation or depolymerization of lignin prior to its incorporation in PF resoles/resins to increase its reactivity by breaking its three-dimensional structure into oligomers of considerably smaller molecular weight ranges. Common de-polymerization techniques include pyrolysis, liquefaction, oxidation, hydrogenation, and hydrolysis. For instance, Kraft lignin and lignosulfonate, both produced as byproducts in the pulping/paper industry were oxidized by different oxidizing agents for use in phenolic resin composites used for the production of abrasive components [11].

Curing temperature is one of the most important parameters in the synthesis of PF resins/resoles. Especially, incorporation of renewable resource such as lignin at a large phenol substitution ratio tends to increase the curing temperature of the phenolic resin primarily due to the increased complexity of the lignin structure when compared to a phenol molecule [12]. In regard to the energy expenditure purpose, lower curing temperatures are preferred. Although the preparation of PF resoles from different feedstocks have been documented, no report is currently available on the synthesis of lignin-based PF resoles from hydrolytically depolymerized KL and investigating the effect of process parameters on the curing temperature of BPF resoles. The objective of this study is to prepare PF resoles from hydrolytically depolymerized Kraft lignin (DKL), and to investigate the effect of *M_w_* of DKL, percent substitution of phenol-to-lignin, and formaldehyde-to-phenol ratio using Box-Behnken Design (BBD) to find the optimum curing temperature for BPF resoles without compromising the properties of resoles.

## 2. Results and Discussion

### 2.1. Characteristics of KL and DKL

Kraft lignin (KL) was chemically modified via the depolymerization process, as described in the methodology section, before being used for the preparation of BPF resoles. GPC was used to investigate the molecular weights of original KL and DLs. From GPC measurements, the average *M_w_* of original KL was 10,000 g/mol with polydispersity index (PDI) of 2.0. After depolymerization at different reaction conditions, the *M_w_* of lignin decreased to 800, 1200 and 1700 g/mol with narrower PDIs. These results suggest that the depolymerization process effectively cleaved the intermolecular bonds, such as ether bonds, which decreased the molecular weight of lignin, increased the contents of total hydroxyl groups in DL and hence greatly promoted the reactivity of the DL for the preparation of resoles. Given the limited or no research work on the impact of lignin *M_w_* on the properties of BPF resins, the *M_w_* of DL products is of interest in this research project, where it is used as one of the process parameters to produce BPF resoles at high phenol substitutions. The relative *M_w_* of the three DL products were obtained and listed in Table 1, along with the DL yields from the hydrolysis experiments at various severities.

#### 2.1.1. GPC-UV Derived Relative *M_w_* and Percent Yield of DKL Products

Table 1, shows the different reaction conditions for the hydrolysis of KL along with the yields and *M_w_* of the resulting DLs. As the treatment severity increase, the *M_w_* of DLs reduced because the depolymerization reactions are endothermic, higher temperatures were thermodynamically favorable in promoting greater cleavage of aryl-ether linkages within the polymer chains of the lignin molecule [13,14]. 

At the same time the yield of DLs also reduces with increase in treatment severity due to the occurrence of prominent crosslinking reaction lead to solid residues [6,13]. DL with lower *M_w_* can be particularly advantageous in applications for the synthesis of BPF resins, where lignin with lower *M_w_* would result in reduced steric hindrance and provide more phenolic hydroxyl contents, which increases the reactive sites towards formaldehyde, thus facilitating the resinification reactions and enabling production of BPF resins at higher phenol substitutions [13].

#### 2.1.2. Elemental Analysis of KL and DKL Products

The measured carbon, hydrogen, nitrogen, sulfur, and oxygen compositions of the original Kraft lignin (KL), and DKLs obtained from hydrolytic depolymerization reactions for 45 min at 250 and 350 °C are listed in Table 2. CHNS elemental analysis of the DKL products showed that sulfur compounds were markedly reduced by hydrolytic depolymerization of KL.

#### 2.1.3. FT-IR Spectra of DKL Products

**Figure 1:**
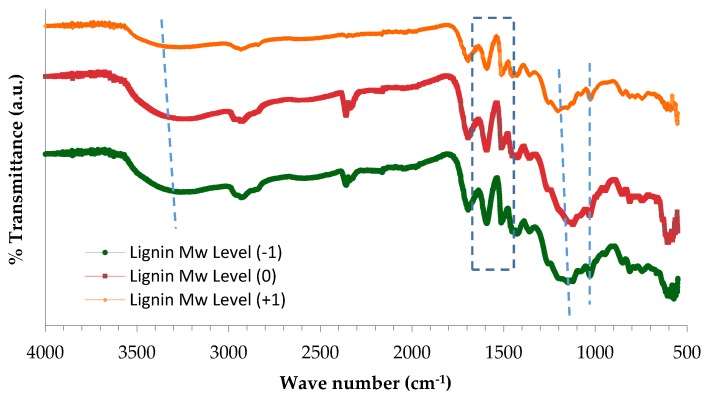
Fourier Transform Infrared (FT-IR) Spectra of DKL products of three levels of *M_w_* (−1), (0), (+1).

Figure 1 shows that absorption bands attributed to -OH stretching appearing at 3280–3320 cm^−1^ for DKLs, partially attributed to the presence of phenolic hydroxyl groups. The absorption bands between 1400 and 1600 can be attributed to C=C aromatic rings while absorption at 1150 indicates the presence of C-O functional groups connected to aromatic rings, which confirm the presence of lignin molecular structure. Weak ether bonds are detected at 1040 cm^−1^ likely as a result of breakage of ether bonds in KL to form hydroxyl groups in DKLs. These results consistent with ^1^H-NMR analysis performed on DKL products by Mahmood et al. [13], where it was found that increases in severity treatment of hydrolytic processing resulted in increases of phenolic hydroxyl groups in the products as compared to the phenolic -OH groups in original KL.

### 2.2. Characterization of Lignin-Based PF Resoles

Table 3 shows the viscosities of all prepared BPF resoles determined with Brookfield Viscometer Model DV-E using spindle 1 at 50 rpm and room temperature. Averages of three measurements were recorded.

Viscosity measurements of phenolic resins provide important information about the advancement of resole resin synthesis and the degree of condensation reactions [15,16]. Low resin viscosities or low degree of condensation reactions may result in over-penetration of adhesive into the wood resulting in starved glue-lines while too high viscosities that may result in low adhesive penetration as well as poor mechanical interlocking of the adhesive into the wood [17]. It can be seen from Table 3 that increasing the phenol percent substitution from 25% to 75% at DL *M_w_* level (−1) and F/P of 2.1 resulted in increase of BPF viscosities from 26 cP to 191 cP. Similarly, increasing the phenol percent substitution from 25% to 75% at DL *M_w_* level (+1) and F/P of 2.1 resulted in increase of BPF viscosities from 124 cP to 348 cP. The increase in viscosity of BPF with higher percent substitutions can be attributed to incorporation of greater lignin content in the BPF resole synthesis. A larger addition amount of lignin or bio-phenolic oils (at a high *M_w_*) likely increases the molecular weight of the resin resulting in higher end viscosities for BPF resoles at 75% phenol-to-lignin substitutions [16]. Research findings from Cheng et al. confirm similar trend of increasing viscosities of bio-based phenolic resins with increasing phenol substitutions [18].

Table 3 also shows that increasing the F/P molar various BPF resoles resulted in reduced BPF viscosities. This seems to be counterintuitive because increasing the F/P ratio increases the degree of methylolation as well as the branching of the BPF polymer, which should result in an increase of viscosity of the BPF resoles. However, this effect might be due to the larger water content that were added into BPF resoles with higher F/P ratios in the form of formalin, an aqueous solution of only 37% formaldehyde. This is actually confirmed by the measurements of pH and non-volatile content for the corresponding resoles. The corresponding pH value and non-volatile content both decrease if increasing F/P molar ratios likely due to the increased amount of water content in the resulted BPF resoles.

Additionally, as *M_w_* of DL increased from level (−1) to level (+1), the viscosities of BPF resoles also increased (Table 3). This trend was consistent for the BPF resoles with 25%, 50%, and 75% phenol substitutions and BPF resoles synthesized at F/P ratios of 1.2 and 3. Using higher *M_w_* of DL in the synthesis of lignin-based phenol formaldehyde resins will likely result in BPF resin with higher molecular weights and increased viscosities [18]. Given the complex structure of lignin, higher *M_w_* of depolymerized lignin could result in lengthening of polymeric chains or additional branching within the structure of BPF resoles, which would then significantly increase the viscosities of the BPF resins [16].

### 2.3. Non-Volatile Contents and pH Measurements of BPF Resoles

Table 4, Table 5 and Table 6 show non-volatile content (NVC) of the synthesized BPF resoles at varying phenol percentage substitutions, varying F/P ratios, and varying *M_w_* the DL used, respectively.

**Figure 2:**
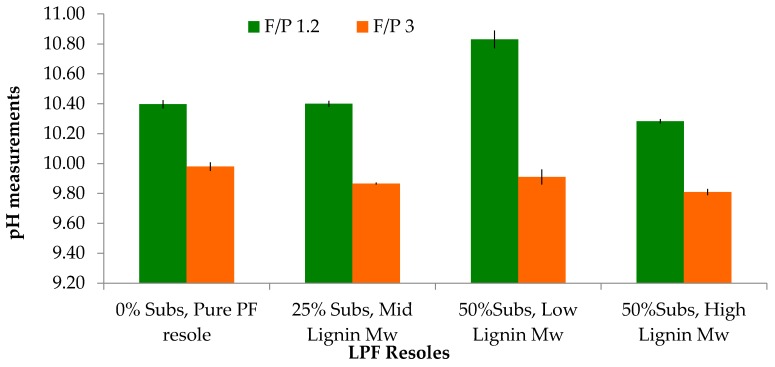
pH of phenolic resoles with varying F/P ratios for pure PF resoles and BPFs at 25% and 50% phenol substitutions.

As shown in the above (Table 4, Table 5 and Table 6), the NVC of BPF resoles synthesized (26–44 wt.%) are normally lower than the recommended NVC range for PF resins as plywood adhesives (i.e., 40–45 wt.%) [8]. To increase the NVC, less water or ethanol content can be used as the solvents during the synthesis of BPF resoles, or the reaction period can be further increased to 3–4 h. However, care must be taken not to cause drastic increases in the viscosities of BPF as a result of potential polymer gelation. Figure 2 clearly indicates that the pH of BPF resoles decreases with rise in F/P molar ratios for pure PF resins as well as BPF resoles at 25% and 50% phenol substitutions. This observation can be attributed to an increase in Cannizarro reactions with higher F/P ratios, whereby formaldehyde undergoes self-condensation reactions and reduces the pH of the resoles. As discussed earlier, higher water content with increasing F/P ratios likely occurred due to increased formalin input. This also explains the increased NVC of BPF resoles with increasing F/P ratios (Table 6).

### 2.4. Curing Temperature of BPF Resoles

**Figure 3:**
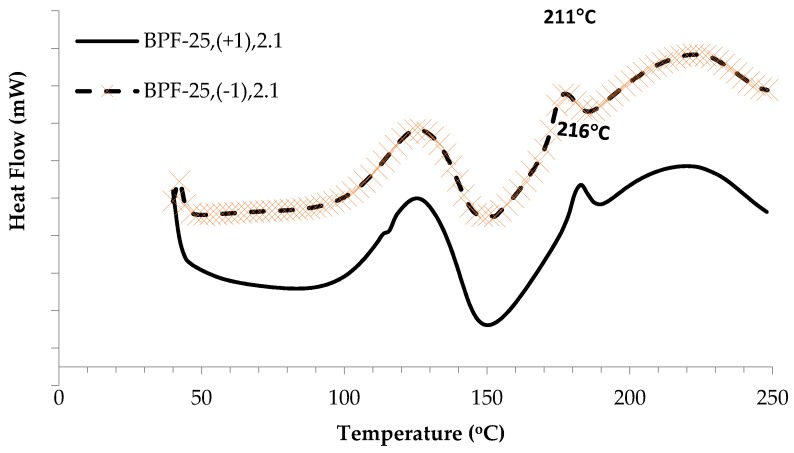
Effects of lignin molecular weight on curing temperature of the BPF Resins at 25% phenol substitution.

**Figure 4:**
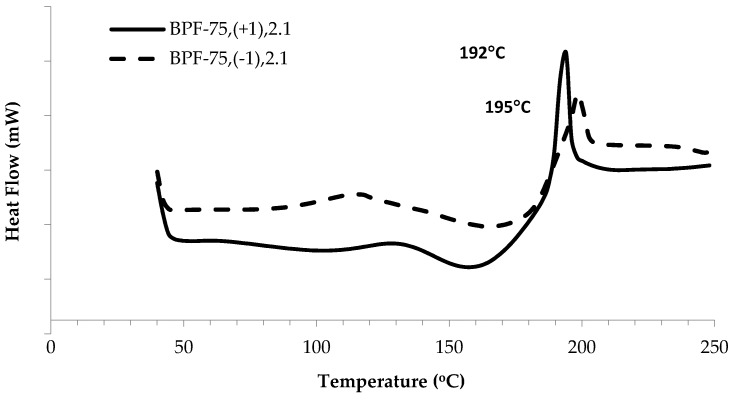
Effect of lignin molecular weight on curing temperature of BPF resins at 75% phenol substitution.

Figure 3 and Figure 4 show the effects of DKL *M_w_* on BPF curing temperature. As can be seen, there were two or three exothermic peaks in the thermograms of the BPF resoles, where the first peak occurred in the temperature range of 115 °C to 135 °C while the second and third peak occurred in the temperature range of 140 °C to 230 °C. The first exothermic peak might be due to addition reactions of free formaldehyde to phenolic rings while the second and third exothermic peaks could be attributed to curing reactions of methylolphenols and phenols to form methylene bridges and condensation reactions of two methylol groups to form dimethylene ether bridges [4].

As can be observed from Figure 3, for BPF resins at a phenol substitution rate of 25%, the BPF synthesized with lower *M_w_* DKL has a slightly lower curing temperatures. It suggests that for BPF resoles at low phenol substitutions, incorporation of lignin with a lower *M_w_* or higher phenolic hydroxyl content at would accelerate the curing rate of the resoles [18]. In contrast, as revealed by Figure 4, the curing temperature of BPF resins with phenol substitution of 75%, the BPF resole prepared with high *M_w_* DKL has a lower curing temperature, which could be explained by the fact that the presence of higher molecular weights bio-phenols with a great level of cross-linking in the resole could be cured readily with a lower the activation energy [19].

### 2.5. Model Building and Analysis of Variance (ANOVA)

The curing temperature (response) from all the synthesis runs based on the experimental design was measured and the results are provided in the Table 7. These results show that curing temperature change with the variation of the synthesis conditions. 

**Figure 5:**
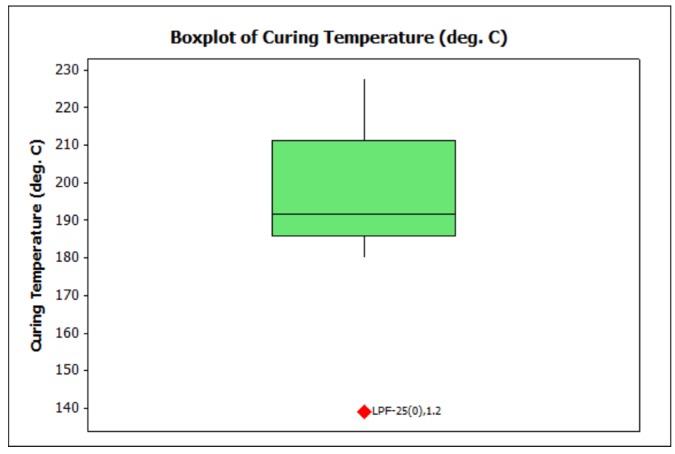
Box plot of curing temperature (°C).

The BBD was applied to optimize the curing temperature for BPF resoles and probe the relative or interaction effects of phenol substitution (%), *M_w_* (g/mol) and F/P ratio on the curing temperature. In this research work, a total of 15 BPF resoles were prepared with three central points and three factors (N = (2) (3) (3 − 1) + 3 = 15). Following the design of the experiments (Table 7), the resoles were synthesized and the curing temperatures were determined using DSC in dynamic mode from 40 °C to 350 °C at 10 °C/min under 50 mL/min of nitrogen gas. Using Minitab, the regression coefficients were estimated and a mathematical model for predicting the curing temperature was built and used to predict the response. The model was then tested for adequacy using analysis of variance (ANOVA) and also interpreted using surface and contour plots. The response (curing temperature) was determined using DSC. All of the DSC thermograms depicted at least two peaks; the first appeared in the temperature range 115 °C and 135 °C while the second appeared in the temperature range 140 °C–230 °C. The one exception was BPF-25,(0),1.2 (see Experimental run ‘1’ in Table 7), where only one peak appeared at 139 °C. A box-plot of the response (curing temperature) in Figure 5 shows that the curing temperature for BPF-25,(0),1.2 was an outlier. Thus, this data point was excluded from the model building.

Using Minitab, the following regression model of response was built (using coded analysis):

T = 185.55 − 9.68 X_1_ − 14.04 X_3_ + 17.77 X_2_^2^ + 6.39 X_3_^2^ + 16.49 X_1_X_3_ − 4.34 X_2_X_3_ (1)

where X_1_ is the substitution of phenol with lignin, X_2_ is the weight average *M_w_* of lignin (g/mol); X_3_ is the formaldehyde-to-phenol ratio; X_2_^2^ and X_3_^2^ are the quadratic terms for second and third variables respectively; and X_1_X_3_ and X_2_X_3_ are the interaction terms. The regression model had the following coefficient of multiple determinations (as determined by Minitab):

R^2^ = 98.05%; R^2^_pred_ = 94.04; R^2^_adj_ = 95.77% (2)

Therefore, ~98% (R^2^) of the variability in curing temperature is accounted for by the model above. R^2^_adj_ was used to ensure that the model is not over-fitted. The R^2^_pred_ of ~94% indicates that the model seems to predict responses to new observations very well. The statistical significance of the coefficients, regression, and residual errors was checked using the *F*-test and the *p*-value test as shown in Table 8 and Table 9.

**Figure 6:**
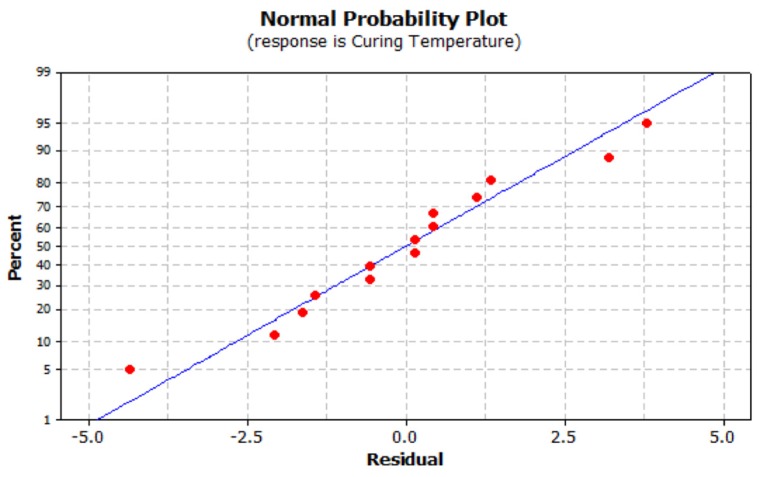
Normal probability plot for curing temperature (response).

**Figure 7:**
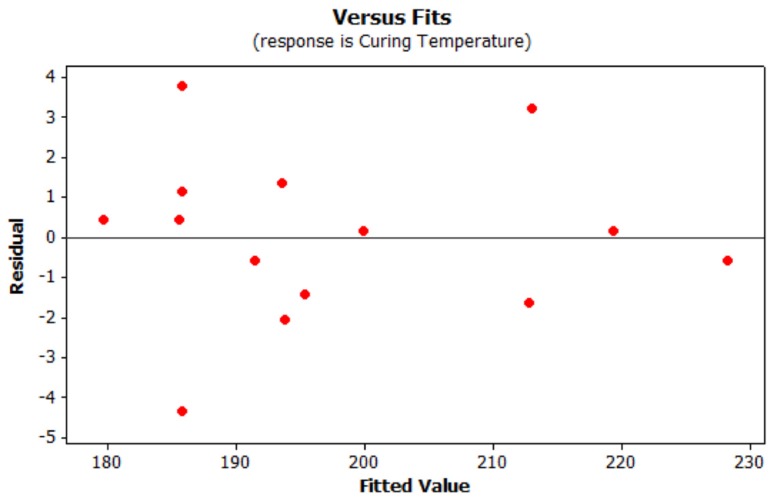
Residual vs. fitted values of the regression.

As can be seen from Table 8, three coefficients were not significant based on a confidence interval of 95% where the corresponding *p*-values ≥ 0.05 including X_2_, X_1_^2^, and X_1_X_2_. Thus, these coefficients were not included in the regression model. From the ANOVA in Table 9, it can be seen that the estimator of variance, mean squares is 9.42 indicating that the regression model is significant. The normal probability plot (Figure 6) indicates that assumption that the errors are normally distributed was a good prediction. The absence of a visible pattern in the residual versus fitted plot (Figure 7) indicates model adequacy.

**Figure 8:**
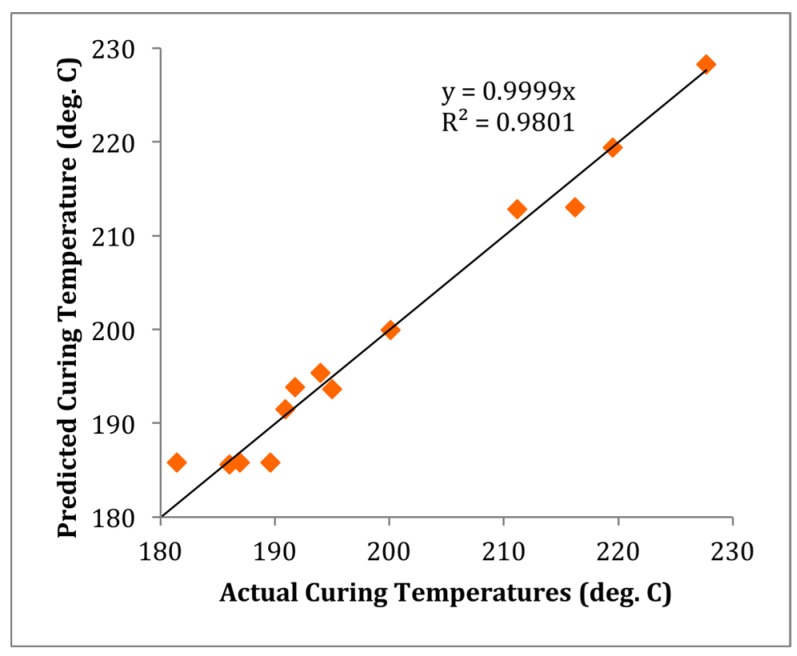
Predicted vs. actual curing temperatures.

Figure 8 shows the actual curing temperatures of the BPF resoles plotted against the predicted curing temperatures from the model. As can be seen, there was an agreement between the predicted data and the actual data for the curing temperature.

### 2.6. Interaction Effects

**Figure 9:**
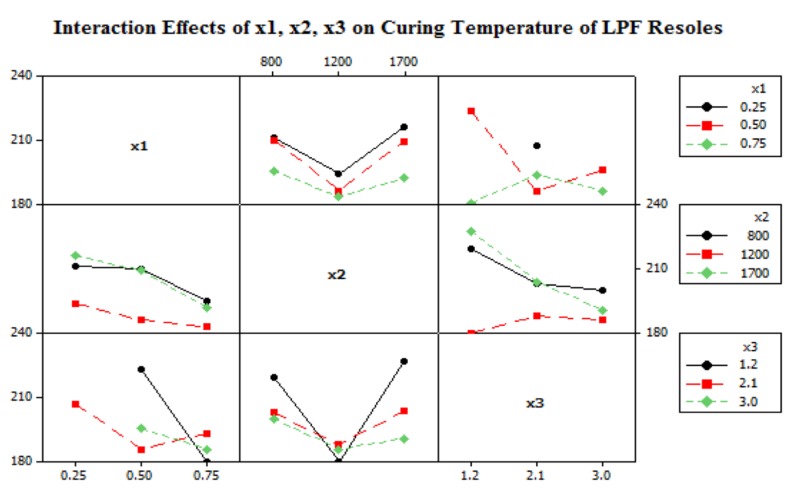
Interaction effects on curing temperature of BPF resoles.

Figure 9 shows the interaction effects of the regression model, where the plots for means of each level of a factor are given while keeping the second factor constant. Absence of parallel lines indicates that the response of one variable depends upon the levels of other variables. As such, assessment of the effect of any of the variables (substitution of phenol with lignin, Lignin *M_w_*, and formaldehyde-to-phenol ratio) should be done with respect to the other variables.

### 2.7. Response Surface Plots and Optimization of Variables

#### 2.7.1. Effect of F/P and Lignin *M_w_* at Constant Percent Substitutions (25%, 50%, 75%) on Curing Temperatures

**Figure 10:**
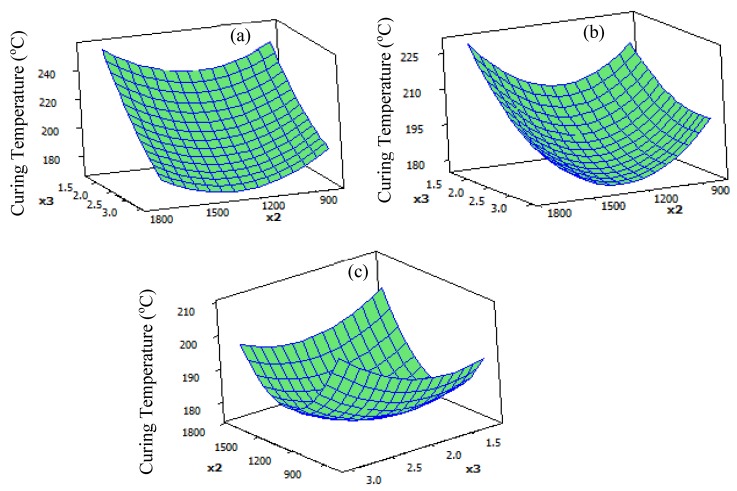
Surface plots for effects of lignin *M_w_* and F/P on curing temperature of BPF resoles at substitution of phenol with lignin at (**a**) 0.25; (**b**) 0.50; (**c**) 0.75.

**Figure 11:**
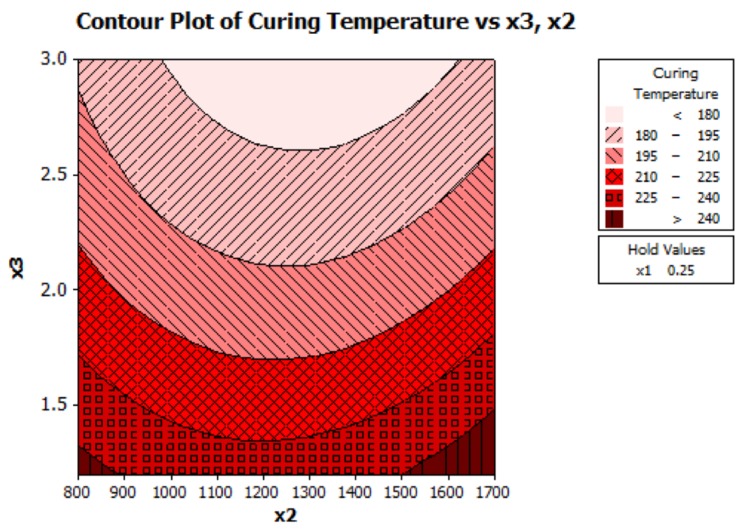
Contour plot for lignin *M_w_* (X_2_) and F/P (X_3_) on curing temperatures of BPF resoles at 25% substitution of phenol with lignin.

Figure 10 shows the surface plots and contour plots (respectively) of lignin weight average molecular weight (X_2_) and F/P molar ratio (X_3_) on the curing temperature when held constant at substitution percentage of (a) 25%; (b) 50%; and (c) 75%. As can be seen from Figure 10a and Figure 11, at 25% substitution of phenol with lignin, lowest curing temperature range occurs at an F/P ratio of slightly higher than 2.5 for lignin *M_w_* of ~1200 g/mol and at F/P ratio of 3 for lignin *M_w_* in the range of ~1000 to ~1600 g/mol. Lower F/P ratios result in higher curing temperatures particularly at highest and lowest ranges of lignin *M_w_*.

**Figure 12:**
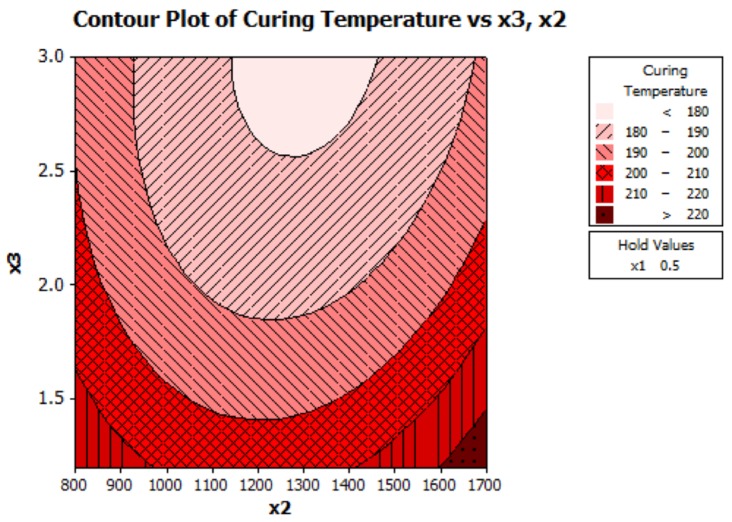
Contour plot for lignin *M_w_* (X_2_) and F/P (X_3_) on curing temperatures of BPF resoles at 50% substitution of phenol with lignin.

**Figure 13:**
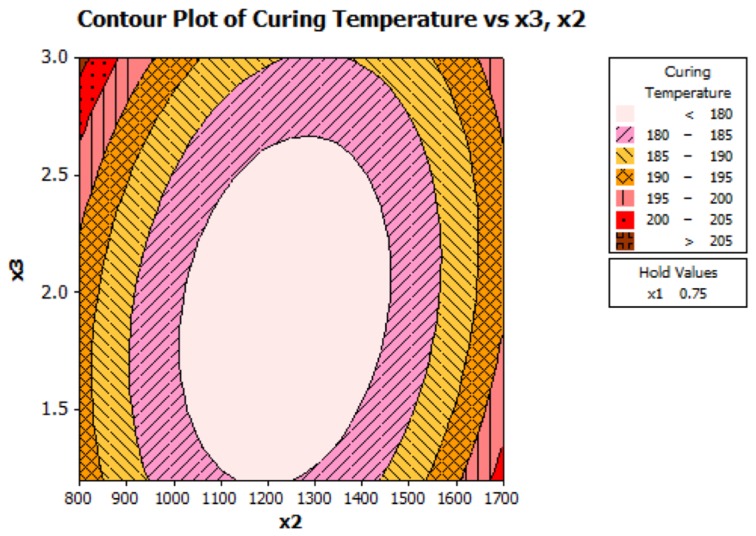
Contour plot for lignin *M_w_* (X_2_) and F/P (X_3_) on curing temperatures of BPF resoles at 75% substitution of phenol with lignin.

A similar trend can be seen (Figure 10b and Figure 12) when the contour plot is held constant at 50% substitution of phenol with lignin; however, the lowest curing temperatures occurs at a narrower range of lignin *M_w_* especially at F/P ratio of 3 (~1100–1450 g/mol). At 75% substitution of phenol with lignin (Figure 10c and Figure 13), the lowest curing temperatures occur at lower F/P ratios while temperature range of 180 °C to 185 °C can occur at F/P ratio of 3 provided that the lignin *M_w_* is ~1250 g/mol. At lignin *M_w_* of less than 900 g/mol and greater than ~1550 g/mol, the curing temperature increases at varying rates depending on the F/P ratios when held constant at 75% substitution of phenol with lignin.

#### 2.7.2. Effect of Percent Substitutions and Lignin *M_w_* at Constant F/P Ratios (1.2, 2.1, 3) on Curing Temperatures

**Figure 14:**
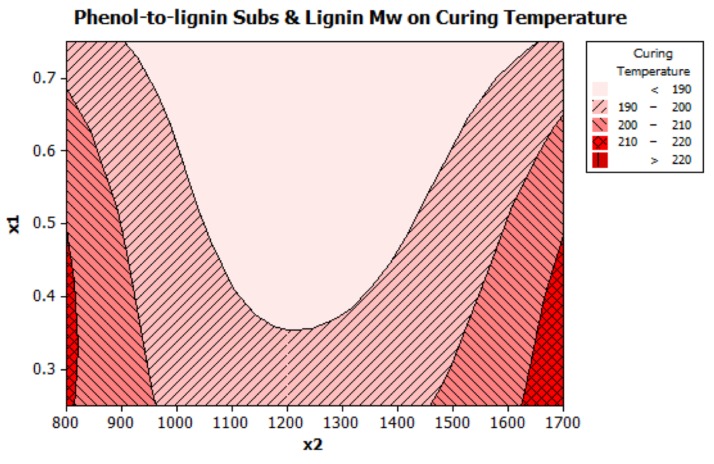
Contour plot for substitution of phenol with lignin (X_1_) *M_w_* on curing temperatures of BPF resoles.

Figure 14 shows the contour plot for substitutions of phenol with lignin and lignin *M_w_* while holding the F/P ratio constant. As can be seen, the two extremes of lignin *M_w_* do not yield the lowest curing temperatures. At higher percent substitutions, the range of lignin *M_w_* that can yield the lowest curing temperature increase, allowing for more variation in lignin *M_w_* at higher percent substitutions. Lignin of *M_w_* ~1200 g/mol allows for yield of lowest curing temperatures (less than 190 °C) from around 35% substitution of phenol with lignin and up to 75% (based on the data range).

#### 2.7.3. Effect of Percent Substitutions and F/P at Lignin *M_w_* (800 g/mol and 1700 g/mol) on Curing Temperatures

**Figure 15:**
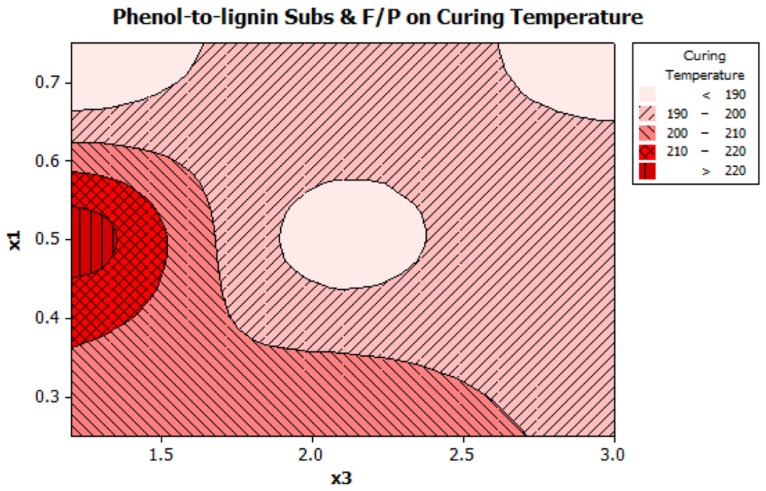
Contour plot for substitution of phenol with lignin (X_1_) and F/P (X_3_) on curing temperatures of BPF resoles.

Figure 15 shows the contour plot for the substitution of phenol with lignin (X_1_) and F/P ratio (X_3_) on the curing temperature while keeping the lignin *M_w_* constant. As can be seen, lowest curing temperatures of less than 190 °C occur at the centre of the two variables as well as at extremes of F/P ratio and high substitution percentage. Therefore, to attain BPF resoles with high percent substitutions, BPF resoles must be synthesized either with low F/P ratios or high F/P ratios. Although low F/P may be desirable for less free formaldehyde content, other factors also need to be taken into consideration like the adhesive strength and the thermal stability of the BPF resoles at both F/P ratios.

**Figure 16:**
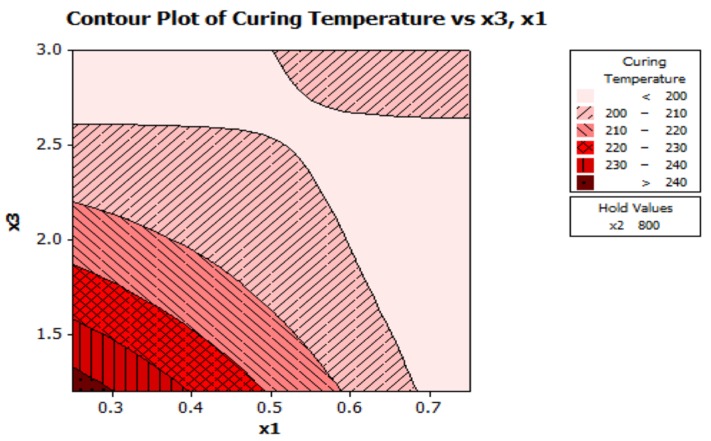
Contour plot for substitution of phenol with lignin (X_1_) and F/P (X_3_) on curing temperatures of BPF resoles at lignin *M_w_* of 800 g/mol.

**Figure 17:**
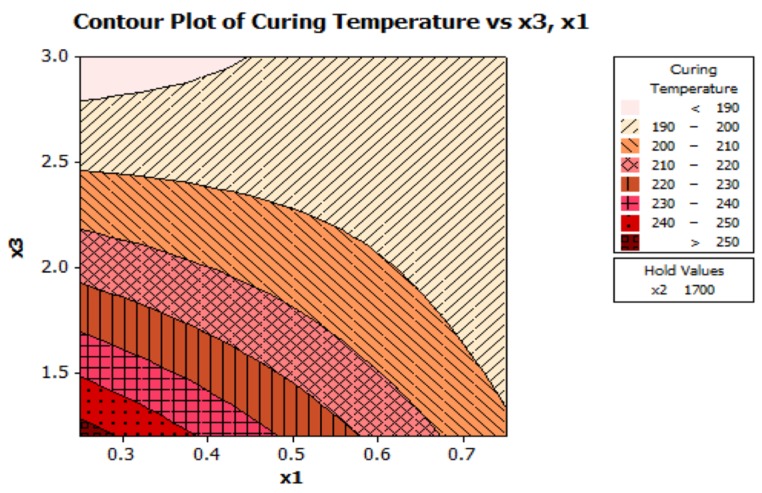
Contour plot for substitution of phenol with lignin (X_1_) and F/P (X_3_) on curing temperatures of BPF resoles at lignin *M_w_* of 1700 g/mol.

At low lignin *M_w_* (~800 g/mol), it can be seen that for high percent substitution, increasing the F/P ratio yields in low curing temperatures of less than 200 °C until around F/P ratio of 2.5 where the curing temperature range increase slightly (Figure 16). At low lignin *M_w_* (~800 g/mol and at lower percent substitutions, higher F/P ratios are preferred. Figure 17 shows that the contour plots for substitution of phenol with lignin and F/P ratio when lignin at higher *M_w_* (~1700 g/mol) is held constant. It can be seen that curing temperatures lower than 190 °C can be attained for BPF resoles synthesized at low percent substitutions and high F/P ratio. However, curing temperatures in the range of 190–200 °C can be still being attained for higher percent substitutions and higher lignin *M_w_* as long as the F/P ratio is higher than 1.2. For both high and low lignin *M_w_*, curing temperatures start to increase when substitution percentage and F/P are decreased.

## 3. Materials and Methods

### 3.1. Materials

Softwood Kraft lignin (KL) used in this study was provided by FPInnovations-Thunder Bay Bioeconomy Technology Centre, in a light brown powder form. The *M_w_* of KL is 10,000 g/mol (polydispersity index ≈ 2.0) based on our GPC-UV analysis. The dried sample contains 0.11 wt.% ash and 63.8 wt.% C, 5.4 wt.% H, 0.02 wt.% N with 5.2 wt.% sulfur (on dry and ash free basis). Other chemicals used in this study, including NaOH (96%), sulfuric acid (99%), acetone (99.5%), ethanol, formaldehyde (37% formalin), solid phenol crystals (99%), tetrahydrofuran (THF, HPLC grade), are all CAS reagent grade chemicals purchased from Sigma-Aldrich (Oakville, ON, Canada) and were used as received.

### 3.2. Hydrolysis of Kraft Lignin

The hydrolytic depolymerization of KL operations was carried out in a 500 mL Model 4848 autoclave reactor (Parr Instruments, Moline, IL, USA) equipped with a pressure gauge, stirrer, thermocouple, gas line, and sampling line. The reactor was charged with 48 g lignin and 60 g distilled water and 132 g NaOH solution (10 wt.%). The reactor was purged thrice with nitrogen, checked for leaks and finally pressurized to 2 MPa. The reactor was then heated at approx. 10 °C/min to the desired temperatures for pre-specified residence times under stirring (290 rpm) according to designed experiments by BBD. During the reaction, the pressure of the reactor system will increase depending on the reaction temperature mainly due to the water vapor pressure (e.g., 5 MPa at 250 °C, 8 MPa at 300 °C up to 16 MPa at 350 °C).

After the pre-set reaction time elapsed, the reactor was immediately quenched with water to stop the further reaction. The gas was released into the fume hood. The reactor contents were then completely rinsed into a beaker using distilled water. The pH value of the washed reactor contents (varying from 11.0 to 9.5 depending on the reaction conditions) was adjusted to approximately 2.0 using 1.0 M H_2_SO_4_ solution to precipitate the DL products. The acidified reaction mixture was then filtered through a Buchner funnel. The solid cake containing depolymerized KL was dissolved in acetone (20–25 mL) under sonication and then filtered under vacuum with Buchner funnel to get acetone soluble depolymerized lignin (DL) or polyols and solid residues (SR). The SRs were dried at 105 °C for 24 h in an oven and weighed to obtain SR yield as wt.% of the original KL on a dry basis. The acetone soluble filtrate was transferred to a pre-weighed Erlenmeyer flask to remove acetone with rotary evaporator at 60 °C followed by 24 h drying in a vacuum oven to obtain the DL products. The yield of DL was calculated based on the mass of original KL on dry basis.

### 3.3. Experimental Design

The process optimization was carried out to optimize the curing temperature of BPF resoles using Box-Behnken design (BBD) with three independent variables. To total 15 experiments were conducted for the preparation of BPF resoles at varying formaldehyde-to-phenol (F/P) ratios, phenol percent substitutions, and DL *M_w_* across three levels. The variables and their levels selected for this study are shown in Table 10. In Table 10 phenol substitution ratio is for phenol-to-lignin ratio and F/P ratio stands for the formaldehyde-to-phenol ratio. The actual values of factors were coded using the equation: xi = (X_i_ − X_i0_)/X_i_, where xi is coded value for the ith independent variable, xi0 is the actual value of the i^th^ independent variable at the centre point, and X_i_ is the step change value [20]. The experiments were designed based on a three-factor Box Behnken Design (BBD) as shown previously in Table 7.

The Box-Behnken design is a three-level partial factorial design where experiments are statistically designed to have treatment combinations that are at the midpoints of the edges of the experimental space. In statistics, BBD is an experimental design for response surface methodology, devised by George E.P. Box and Donald Behnken in 1960. The main aim of the surface response methodology (RSM) is to optimize the responses [21]. The total numbers of experiments follow the equation:

N = 2k (k − 1) + C0(3)

where k is the number of parameters, factors, or variables, and C0 is the number of central points.

### 3.4. Synthesis of BPF Resoles

Various BPF resoles were synthesized with DL of different *M_w_* values, phenol percent substitutions, and F/P molar ratios, according to the Box-Behnken Design (BBD) of experiment Design (Table 7). The resinification was carried out in a 250 mL three-neck flask equipped with a thermometer, a pressure-equalizing addition funnel, and a condenser and then placed over a water-bath on top of a hot plate. The mixture of phenol, DL, sodium hydroxide (NaOH), ethanol and water were added at pre-determined amounts into the three-neck flask. The required amounts of phenol and lignin were determined by the phenol percent substitution ratio for the experimental run from the BBD. Sodium hydroxide solution and water were added at concentrations of 10 wt.% and 40 wt.% based on phenolic compounds, respectively, referring to the literature study [22].

**Scheme 1:**
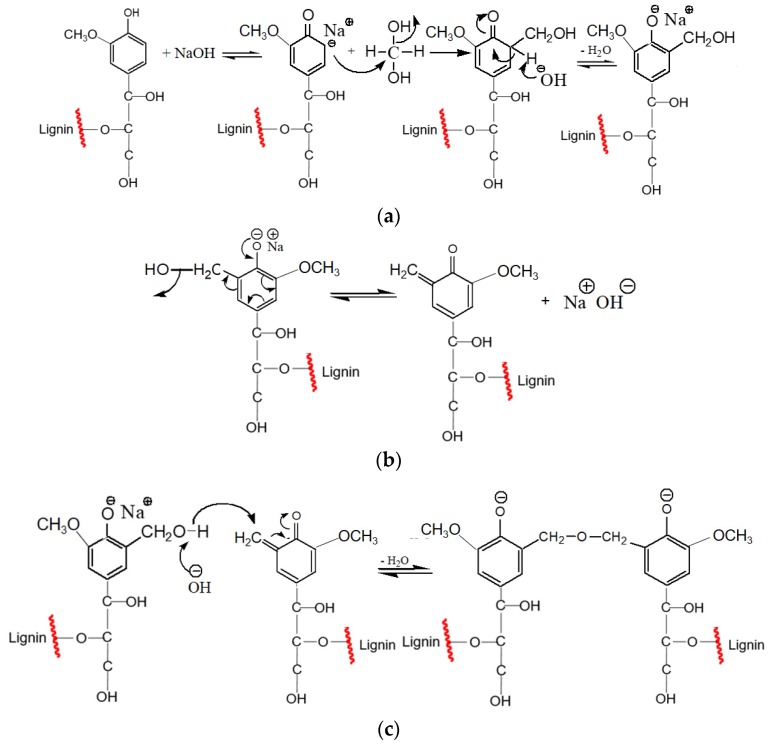
The proposed reaction mechanism of lignin-based bio-phenol formaldehyde resin formation. (**a**) Formation of monosubstituted methylolated phenols through addition reaction; (**b**) Formation of quinone methide intermediates; (**c**) Formation of diphenyl-methylene.

As well known, lignin as well as de-polymerized lignin are natural macromolecular molecules. By far the detailed mechanism of lignin-based bio-phenol-formaldehyde formation is yet to be elucidated. However, analogy to the mechanism of conventional phenol-formaldehyde resin formation [22], the schematic reaction mechanism of lignin-based bio-phenol formaldehyde resin formation can be proposed and illustrated in Scheme 1:

Ethanol was added to improve the solubility of DL at a mass equal to that all phenolic compounds including pure phenol and DL. To avoid the formation of coagulants during the reaction especially at high percent phenol substitutions, sodium hydroxide solution was mixed with phenol and added drop wise. The mixture was allowed to mix for a period of two hours under magnetic stirring at 60 °C to ensure a homogenous lignin-phenol solution.

The reaction temperature was then increased to 80 °C and formalin (37% formaldehyde by weight) was added drop-wise into the three-neck flask using a cylindrical separator funnel 9 given that the reaction is exothermic. The amount of formaldehyde added was determined by the required F/P molar ratio of the experimental run, where P denotes all phenolic compounds including phenol and DL (assuming *M_w_* of phenolic compounds to be 94.11 g/mol to calculate F/P ratios). To minimize the free formaldehyde content of BPF resins [18], the reaction was held at 80 °C for 2 h, and then stopped by cooling to room temperature. The resultant BPF resin was then recovered into labeled 250 mL plastic bottles and stored in the freezer at 2 °C for further characterization purposes. The synthesized BPF resins were labeled as “BPF-25,(+1),2.1”, denoting BPF-, % phenol substitution, (the DL *M_w_* level), F/P ratio. For example, BPF-25,(+1),2.1 indicates that the BPF has phenol substitution ratio of 25% with DL of the highest *M_w_* level and was synthesized using F/P ratio of 2.1. In a typical resinification experiment, e.g., to produce 150 mL of BPF-50,(0),2.1, 18 g of DL at *M_w_* level of (0) were allowed to mix with 18 g of phenol, 36 mL of ethanol and 14.4 g of distilled water followed by an addition of 65.2 g of formalin for F/P molar ratio of 2.1:1.

### 3.5. Analysis and Characterization

The relative molecular weights (*M_w_* and *M_n_*) of DLs were measured with a Breeze GPC-HPLC instrument (Waters, Mississauga, ON, Canada), 1525 binary pump, UV detector set at 270 nm, Waters Styragel HR1 column at 40 °C) using THF as the eluent at a flow rate of 1 mL/min with linear polystyrene standards for the molecular weight calibration curve). The elemental compositions of carbon, hydrogen, nitrogen, and sulfur in KL and DKL were analyzed using a CHNS Flash Elemental Analyzer 1112 series (Thermo Scientific, Waltham, MA, USA) with helium as a carrier gas. The functional groups of DKL were analyzed using Fourier Transform Infrared (FT-IR) Spectroscopy (TGA-FTIR coupled system, Perkin-Elmer, Waltham, MA, USA). Viscosity measurements of the synthesized BPF resoles were taken using Digital Viscometer Model DV-E (Brookfield Engineering, Middleboro, MA, USA) at 50 rpm and room temperature in accordance to recommendations by ISO-2555 for plywood adhesives. An average of three measurements were taken and recorded for each BPF resole. The pH measurements of final BPF resole products were taken using a digital pH probe at room temperature. The nonvolatile contents of BPF resoles were determined in accordance to ASTM D4426-01 (2006). Around 1 g of each BPF sample was weighed on a glass dish, and heated in an oven at 125 °C for 105 min. Next, the BPF sample was placed in desiccators for a period of 5–15 min and then weighed again to determine the weight percentage of the residues in relation to the original liquid BPF sample, i.e., the non-volatile content (NVC). To investigate the thermal curing properties of the synthesized BPF resoles, Differential Scanning Calorimetry (DSC) (DSC1, Mettler-Toledo, Columbus, OH, USA) was used to test dried resin samples in sealed aluminum crucibles between temperatures of 40 °C and 350 °C at a heating rate of 10 °C/min under nitrogen gas at 50 mL/min. From the obtained DSC thermograms, the curing peaks of BPF resoles were determined and recorded.

It shall be noted that although the free formaldehyde contents of the synthesized BPF resins were not measured, the method used in this work for synthesizing the BPF resins was adopted from a previous work of our research group by Cheng et al. [18], where the free formaldehyde content in depolymerized lignin based PF resins with a phenol substitution ratio of 50–75% was measured in the range of 0.5–0.7% due to the relatively lower reactivity of bio-phenols (de-polymerized lignin). According to the research findings from the optimization study as reported in this work, BPF resins with lower F/P ratios can be synthesized, thereby ensuring minimal free formaldehyde content and making the process more cost effective.

## 4. Conclusions

A regression model was successfully built for the prediction of curing temperature using the synthesis parameters substitution of phenol with lignin, average weight molecular weight of lignin (*M_w_*) and formaldehyde to phenol ratio. It was verified for its fit and adequacy using the coefficients of determination, analysis of variance (ANOVA), significance test of coefficients, normal probability plot, residuals versus fitted data plot, and the predicted versus actual plot. Contour and surface plots indicate that lignin average weight molecular weight should be ~1200 g/mol provides low temperatures of less than 180 °C at any substitution percentage. Studies have indicated that the incorporation of lignin can reduce the curing temperature of the resin because it allows for larger molecules to make contact and hence enhances entanglement of final resole network (Rodriguez, 1996). However, at higher lignin *M_w_*, steric hindrance effects may start to hinder the cross-linking reaction rates. For both high lignin *M_w_* and low lignin *M_w_*, curing temperature increases with lower F/P ratios and lower substitution of phenol-to-lignin. At 25% and 50% substitution of phenol with lignin, increasing the F/P ratio allows for synthesis of resins with low curing temperatures. Increasing the substitution of phenol-to-lignin allows for a broader range of lignin weight average molecular weight to attain low curing temperatures.

## Figures and Tables

**Table 1:** GPC-UV derived relative *M_w_* and percent yield of the three depolymerized lignin levels.

*M_w_* of DL	Reaction Temp. (°C)	Residence Time (min)	*M_w_* (g/mol)	DL Yield (wt.%)
Level (−1)	250	45	1700	83.3 ± 3.7
Level (0)	300	60	1200	71.1 ± 1.7
Level (+1)	350	45	800	31.3 ± 2.4

**Table 2:** Elemental compositions of KL and DKL products obtained from hydrolytic depolymerization reactions at various severities corresponding to different levels (−1 and +1) of *M_w_*.

KL or DKL Level	Elemental Composition				
	C	H	N	S	O ^a^
KL ^b^	63.8	5.4	0.02	5.2	25.6
DKL, Level (−1)	63.6 ± 1.8	5.3 ± 0.02	0.07 ± 0.03	1.8 ± 0.11	29.2 ± 0.17
DKL, Level (+1)	75.5 ± 0.16	5.9 ± 0.02	0.11 ± 0.01	0.21 ± 0.04	18.2 ± 0.18

^a^ by difference. ^b^ KL results were taken from Ref. [13].

**Table 3:** Viscosity of all BPF resoles synthesized in this study.

Sample ID	Viscosity (cP)
LPF-25,(−1),2.1	26 ± 1.2
LPF-50,(−1),1.2	229 ± 8.5
LPF-50,(−1),3	165 ± 7.2
LPF-75,(−1),2.1	191 ± 3.5
LPF-25,(+1),2.1	124 ± 0.4
LPF-50,(+1),1.2	288 ± 0.8
LPF-50,(+1),3	137 ± 1.2
LPF-75,(+1),2.1	348 ± 0.6
LPF-25,(0),1.2	124 ± 0.1
LPF-25,(0),3	16 ± 0.1
LPF-50,(0),2.1	40 ± 0.1
LPF-75,(0),1.2	>5000
LPF-75,(0),3	>5000

**Table 4:** NVC of BPF resoles with varying phenol percent substitutions (left to right).

25% Substitution	NVC (wt.%)	75% Substitution	NVC (wt.%)
BPF-25,(−1),2.1	36	BPF-75,(−1),2.1	26
BPF-25,(+1),2.1	34	BPF-75,(+1),2.1	30

**Table 5:** NVC of BPF resoles with varying phenol percent substitutions (left to right).

F/P of 1.2	NVC (wt.%)	F/P of 3	NVC (wt.%)
BPF-50,(−1),1.2	44	BPF-50,(−1),3	33
BPF-50,(+1),1.2	35	BPF-50,(+1),3	28
BPF-25,(0),1.2	35	BPF-25,(0),3	30
BPF-75,(0),1.2	41	BPF-75,(0),3	39

**Table 6:** NVC of BPF resoles with increasing *M_w_* of DL (left to right).

Low *M_w_* of DL	NVC (wt.%)	High *M_w_* of DL	NVC (wt.%)
BPF-25,(−1),2.1	36	BPF-25,(+1),2.1	34
BPF-50,(−1),1.2	44	BPF-50,(+1),1.2	35
BPF-50,(−1),3	33	BPF-50,(+1),3	28
BPF-75,(−1),2.1	26	BPF-75,(+1),2.1	30

**Table 7:** BBD Matrix for the effect of phenol-to-lignin substitution, (X_1_) Lignin *M_w_* (X_2_). F/P molar ratio (X_3_) on the Curing Temperature (°C) of BPF resoles including experimental results.

Sample ID ^1^	Experiment Number	Variable Levels			Response (Curing T) (°C)
		X_1_	X_2_	X_3_	
BPF-25,(0),1.2	1	−1	0	−1	139.27
BPF-25,(0),3	2	−1	0	+1	193.95
BPF-75,(0),1.2	3	+1	0	−1	180.14
BPF-75,(0),3	4	+1	0	+1	185.99
BPF-50,(−1),1.2	5	0	−1	−1	219.50
BPF-50,(−1),3	6	0	−1	+1	200.09
BPF-50,(+1)1.2	7	0	+1	−1	227.66
BPF-50,(+1),3	8	0	+1	+1	190.90
BPF-25,(−1),2.1	9	−1	−1	0	211.17
BPF-75,(−1)2.1	10	+1	−1	0	194.96
BPF-25,(+1),2.1	11	−1	+1	0	216.20
BPF-75,(+1),2.1	12	+1	+1	0	191.75
BPF-50,(0),2.1 (1)	13	0	0	0	189.57
BPF-50,(0),2.1 (2)	14	0	0	0	186.91
BPF-50,(0),2.1 (3)	15	0	0	0	181.44

^1^ Note: Samples are labeled as follows: BPF-% substitution of phenol with lignin, Lignin *M_w_* level, F/P ratio.

**Table 8:** Statistical significance of the coefficient of regression.

Model Term	Parameter Estimate	Standard Error	*T*-Value	*p*-Value
Constant	185.55	1.77	105.03	0.00
X_1_	−9.68	1.30	−7.43	0.002
X_2_	0.10	1.10	0.09	0.933
X_3_	−14.04	1.55	−9.04	0.001
X_1_^2^	0.04	1.78	0.02	0.982
X_2_^2^	17.77	1.76	10.07	0.001
X_3_^2^	6.39	1.94	3.29	0.030
X_1_X_2_	−2.11	1.55	−1.36	0.244
X_1_X_3_	16.49	2.70	6.12	0.004
X_2_X_3_	−4.34	1.55	−2.79	0.049

**Table 9:** ANOVA for the regression.

Source	df	Sum of Squares	Mean Squares	*F*-Value
Regression	7	2840.88	405.84	43.07
Residual	6	56.54	9.42	
Total	13	2897.42		

**Table 10:** Coded Values of the Variables for the Box-Behnken Design.

Parameter	Variable	Level (−1)	Level (0)	Level (+1)
Phenol substitution (%)	X	25	50	75
*M_w_* (g/mol)	X	800	1200	1700
F/P ratio	X	1.2	2.1	3.0
